# KinCor, a national registry for paediatric patients with congenital and other types of heart disease in the Netherlands: aims, design and interim results

**DOI:** 10.1007/s12471-016-0892-9

**Published:** 2016-09-08

**Authors:** L. M. Silva, I. M. Kuipers, F. van den Heuvel, R. Mendes, R. M. F. Berger, I. M. van Beynum, L. Rozendaal, L. A. J. Rammeloo, G. G. van Iperen, M. Schokking, S. Frerich, N. A. Blom, J. M. P. J. Breur, W. A. Helbing

**Affiliations:** 1 Department of Paediatrics, Division of Paediatric Cardiology, Erasmus University Medical Centre, Rotterdam, The Netherlands; 2 Department of Paediatrics, Division of Paediatric Cardiology, Academic Medical Centre, Amsterdam, The Netherlands; 3 Department of Paediatric Cardiology, Beatrix Children’s Hospital, University Medical Centre Groningen, University of Groningen, Groningen, The Netherlands; 4 Health E‑Solutions, Rotterdam, The Netherlands; 5 Department of Paediatrics, Division of Paediatric Cardiology, Leiden University Medical Centre, Leiden, The Netherlands; 6 Department of Paediatrics, Division of Paediatric Cardiology, VU Medical Centre, Amsterdam, The Netherlands; 7 Department of Paediatrics, Division of Paediatric Cardiology, Utrecht University Medical Centre, Utrecht, The Netherlands; 8 Department of Paediatrics, Division of Paediatric Cardiology, Radboud University Medical Centre, Nijmegen, The Netherlands; 9 Department of Paediatrics, Division of Paediatric Cardiology, Maastricht University Medical Centre, Maastricht, The Netherlands

**Keywords:** Registry, Database, Child, Paediatric, Congenital, Heart disease

## Abstract

**Objective:**

Studies in children with heart disease have been hampered by a lack of easily identifiable patient groups. Currently, there are few prospective population-based registries covering the entire spectrum of heart disease in children. KinCor is a Dutch national registry for children with heart diseases. This paper presents the aims, design and interim results of the KinCor project.

**Methods:**

All children presenting at a Dutch university medical centre with a diagnosis of heart disease from 2012 onwards were eligible for registration in the KinCor database. Data entry is through a web-based portal. Entry codes have been synchronised with the European Paediatric Cardiac Coding system, allowing coupling with similar databases for adults, such as CONCOR.

**Results:**

Between June 2012 and July 2015, 8421 patients were registered (76 % of those eligible). Median age of the patients was 9.8 years, 44.7 % were female; 6782 patients had morphological congenital heart disease. The most prevalent morphological congenital heart defects were ventricular septal defects (18 %), Tetralogy of Fallot (10 %) and transposition of great arteries (9 %). For 42 % of the patients additional diagnoses were registered. Sixty percent of patients had undergone at least one intervention (catheter intervention or surgery).

**Conclusion:**

The KinCor database has developed into a large registry of data of children with all types of heart disease and continues to grow. This database will provide the opportunity for epidemiological research projects on congenital and other types of heart disease in children. Entry codes are shared with the CONCOR database, which may provide a unique dataset.

## Introduction

Congenital heart diseases (CHD) are a substantial burden on global health [1] and have major impact on patients’ survival and quality of life [2]. Expressed in disability-adjusted life years, the impact of CHD ranks among the highest of all diseases [3].

Estimates of birth prevalence of CHD vary between countries and through time periods and have ranged from 2.5 to 5.5 per 1000 live births [4, 5]. Information on population-based prevalence rates of CHD in the paediatric age range is scarce and has been shown to range from 6.3 to 13.1 per 1000 children aged 0 to 18 years [6–8]. More up-to-date population-based information on prevalence of heart disease in children is required. Furthermore, there is a strong need for data on outcome and on the clinical state of children with heart disease. Ideally, this should cover the entire age range of this patient population, a wide spectrum of heart anomalies and a high level of inclusion. Population-based registries of patients with heart disease can provide the infrastructure for extensive epidemiological research in this field. In the Netherlands, there is a national registry for adult patients with CHD, CONCOR [9]. However, a national registry for paediatric patients with heart disease has been lacking. KinCor, a Dutch national registry for paediatric patients with heart diseases, was launched in 2012. In this paper, we aim to provide information on the opportunities offered by this project and present interim results, particularly the distribution of the various diagnoses in the database after three years of inclusion.

## Methods

### Organisation

The KinCor project was initiated by the departments of paediatric cardiology of the eight university medical centres in the Netherlands (see author affiliations), in collaboration with the Netherlands Heart Foundation and the Dutch Federation of University Medical Centres.

The general aims of the KinCor project are to facilitate studies on heart disease in children with regard to:

- epidemiology;
- risk factors for mortality and morbidity;
- larger cohort and intervention studies in specific patient populations;
- the effects of different treatment strategies.

A steering committee and a project committee manage the project. The steering committee is responsible for general policy and management of the KinCor project, including strategic, scientific and legal aspects and implementation of the registration system in the participating departments.

The project committee consists of representatives of each of the involved departments and is responsible for actual patient inclusion, data collection, coding, data entry, data management and management of the research nurses.

The research committee consists of paediatric cardiologists and a representative from one of the other paediatric subspecialties, patient organisations and a legal counsellor. This committee is responsible for the evaluation of research protocols and approval of requests for data.

### Ethics and regulations

The KinCor database complies with the national Personal Data Protection Act and has been reported to the Personal Data Authority. Legal compliance has been tested and approved by the contributing centres.

### Study population

All children aged 0 to 18 years presenting at the participating departments with a new or known diagnosis of heart disease are eligible for inclusion in the KinCor database. Patients who meet the inclusion criteria are informed about the KinCor project during an outpatient visit. They are handed a document with detailed information about KinCor, along with a consent form that patients and/or their parents can hand in right away, or later by mail. For additional information patients are also referred to the website (www.kincor.nl). It is only after consent is given that relevant information is retrospectively collected based on medical records, and data on diagnosis, interventions and life status are prospectively collected during follow-up. At each outpatient visit, data of registered patients are updated by the data managers by checking for new diagnoses, interventions, diagnostic procedures and complications. Inclusion and update procedures are evaluated twice a year during meetings for the data managers.

Excluded from inclusion are patients who:

- were discharged from outpatient follow-up before start of the registration,
- were transferred to the care of an adult cardiologist before start of the registration.

Children with heart disease who were exclusively followed in non-academic medical centres have not been included in the KinCor registration so far, but will be in the near future.

Parents or legal guardians are asked for consent for inclusion of their child’s data in the registration system. When the child is 12 years or older, both parents/guardians and the child are asked for consent, in accordance with Dutch legislation.

### Data coding and entry

Diagnoses, surgical interventions, catheter interventions and other interventions are coded using the European Paediatric Cardiac Coding (EPCC) system [10]. For the purpose of the KinCor database, subsets of 76 and 152 codes for diagnoses of heart disease and interventions, respectively, were selected. In case of multiple diagnoses in one patient, the defect or disease that is judged by the attending cardiologist to be predominant is the *main diagnosis. *The other diagnoses are recorded as *subdiagnoses*. If a patient has both CHD and acquired heart disease, the congenital defect is recorded as the main diagnosis. Individual participants are labelled with a unique and anonymised KinCor identifier. For each participant, date of birth, gender, diagnosis and any catheter and/or surgical interventions are entered into the database. Status (alive, deceased, lost to follow-up) and date of diagnosis when heart disease was not diagnosed at birth are also recorded.

### Security

Three levels of security are used to ensure safety of the data in the KinCor database: server security, application security and database security. The KinCor application and database are running on servers hosted by a party certified according to NEN7510, ISO 27001, ISO 9001 and ISO 14001. The NEN7510 and ISO 27001 are standards for information security and are obligatory to ensure safe storage of patient-related data on servers. Along with the security protocols in place to comply with these information security standards there are firewall policies set up to restrict access to the hosting environment containing the data. Within the application, several other measures have been taken to ensure the data’s integrity and safety, such as an SSL encryption layer to reach the frontend of the application, and application coding security to prevent the possibilities of ‘SQL injection’, ‘Cross Site Scripting’, ‘Cross Site Request Forgery’ and ‘Click Jacking’. Furthermore, a two-factor authentication procedure is used which provides identification of users by means of a combination of two different components. The KinCor application uses a username-password combination component and a security token that generates a cryptographic key which is requested upon access of the application. Also, all database transactions are logged.

All data that can be traced back to the identity of the patients are stored in the database with an AES encryption. In case a patient no longer wishes to take part in the KinCor project, the data are anonymised through a SHA224 cryptographic hashing algorithm.

### Quality insurance

At regular intervals a random sample of the data in the database is checked for its compliance with the source data (individual electronic patient files in submitting hospitals) by cross check by members of the project group. In an initial step we checked the data present for seven congenital heart diagnoses in three participating centres. Twenty-one patients were selected per centre. The agreement between the data in the database and in the source files was excellent.

### Requests for data use for research projects

Researchers may submit a request for data from the KinCor database for specific research projects. For details on this procedure please see www.kincor.nl.

### Data analyses

We have assessed the total number of patients registered between June 2012 and July 2015. For the purpose of this article, heart diseases were divided into two categories: *morphological congenital heart diseases*, that is, developmental defects resulting in structural malformations, and *other types of hearts diseases, *including for example cardiomyopathies and conduction and rhythm disorders. All analyses were performed using Statistical Package of Social Sciences (SPSS) version 22.0 for windows.

## Interim results

Between June 2012 and July 2015, 8421 patients were registered in the KinCor database. This corresponds with 76 % of eligible patients and with 95 % of all patients who responded to the request for participation. Median age of the patients was 9.8 years; 44.6 % were female (Table 1). Ninety-one percent of patients were alive and in follow-up at the time of this study. In the last 3 years 68 patients died (data not shown). In 41 cases, death was directly related to a previous intervention or diagnostic procedure (26 patients died within 30 days after that intervention or diagnostic procedure, and 15 patients died longer than 30 days after that intervention or diagnostic procedure).

**Table 1:** Characteristics of patients registered between June 2012 and July 2015 (*n* = 8421)^a^

Characteristics	Percentage/Median (range)
Age (median and range in years)	9.8 (0.15–20.9)
Age group (%)	
0–1 year	2.8
1–4 years	18.3
4–12 years	39.3
12–16 years	22.9
>16 years	16.7
Gender (%)	
Female	44.7
Male	55.3
Status (%)	
Alive and in follow-up	91
Alive at discharge or transfer^b^	5.1
Died at or after birth	0.2
Unknown status or loss to follow-up	3.7

^a^Values are percentages in case of categorical variables, or means (with standard deviation) in case of continuous variables

^b^Alive at discharge from follow-up or at transfer to adult cardiologist or general paediatrician

Of the total population of patients, 6782 (80 %) had a morphological CHD as main diagnosis, and 1639 had another type of heart disease (20 %).

**Fig. 1:**
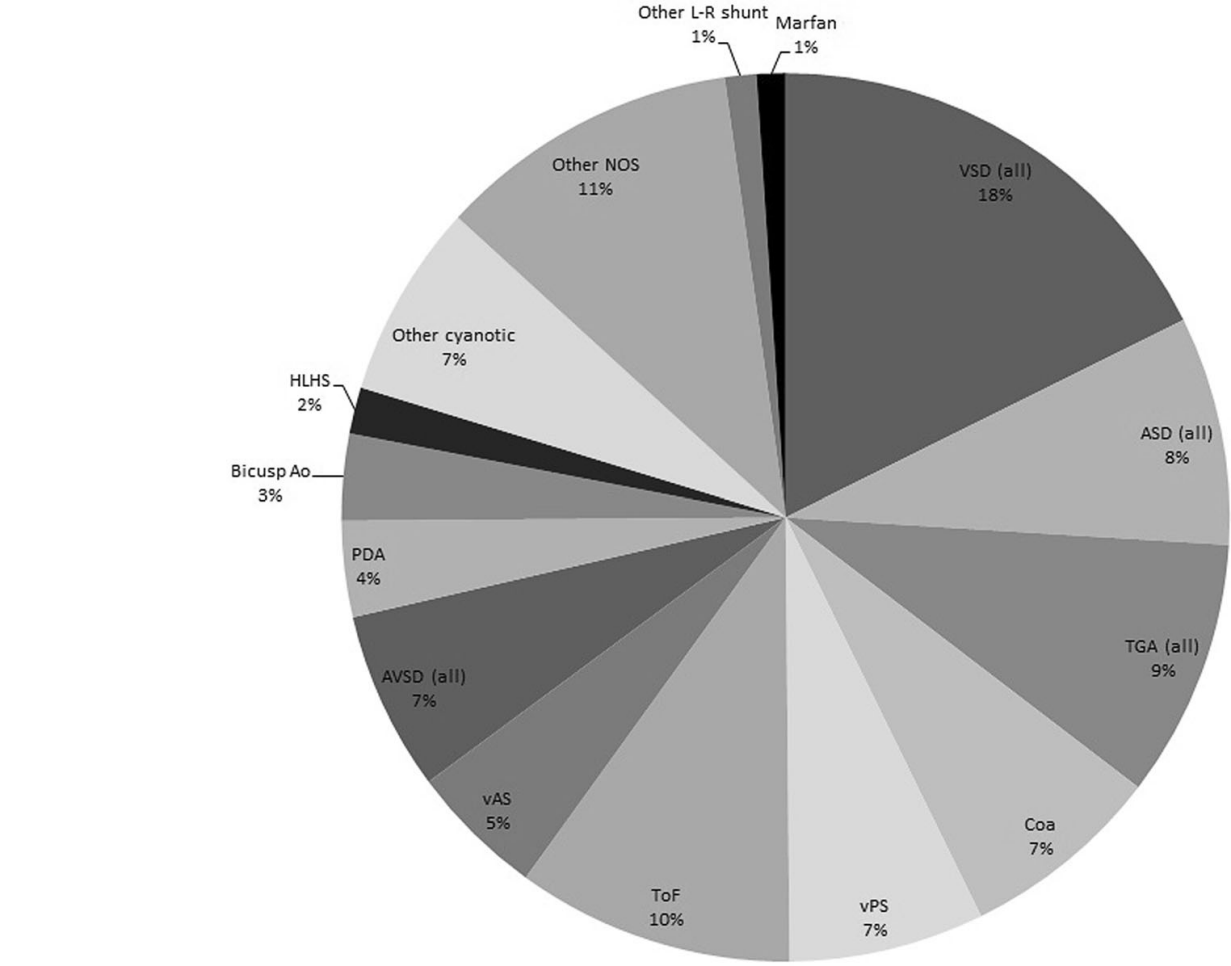
Distribution of morphological congenital heart diseases (*n* = 6782). *VSD* ventricular septal defect, *ASD* atrial septal defect, *TGA* transposition of great arteries, *Coa* coarctation of the aorta, *vPS* valvular pulmonic stenosis, *ToF* Tetralogy of Fallot, *vAS* valvular aortic stenosis, *AVSD* atrioventricular septal defect, *PDA* persistent ductus arteriosus, *Bicusp Ao* bicuspid aortic valve, *HLHS* Hypoplastic Left Heart Syndrome, *NOS* not otherwise specified, *L-R shunt* left to right shunt

**Fig. 2:**
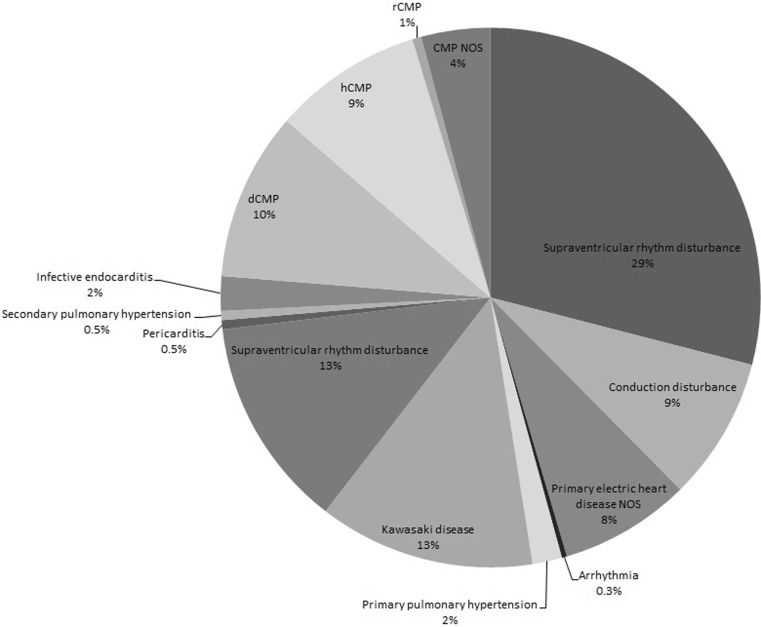
Distribution of other types of heart diseases (*n* = 1639) *dCMP* dilated cardiomyopathy, *hCMP* hypertrophic cardiomyopathy, *rCMP* restrictive cardiomyopathy, *NOS* not otherwise specified

Fig. 1 shows the distribution of morphological CHD registered as main diagnosis. Among these, the most prevalent defects were ventricular septal defects (perimembranous, muscular, subarterial and VSD not otherwise specified; *n* = 1197; 18 %), Tetralogy of Fallot (*n* = 685; 10 %) and transposition of great arteries (*n* = 638; 9 %). Within the category other types of heart diseases, supraventricular rhythm disturbances were by far the most common (29 %; Fig. 2).

**Fig. 3:**
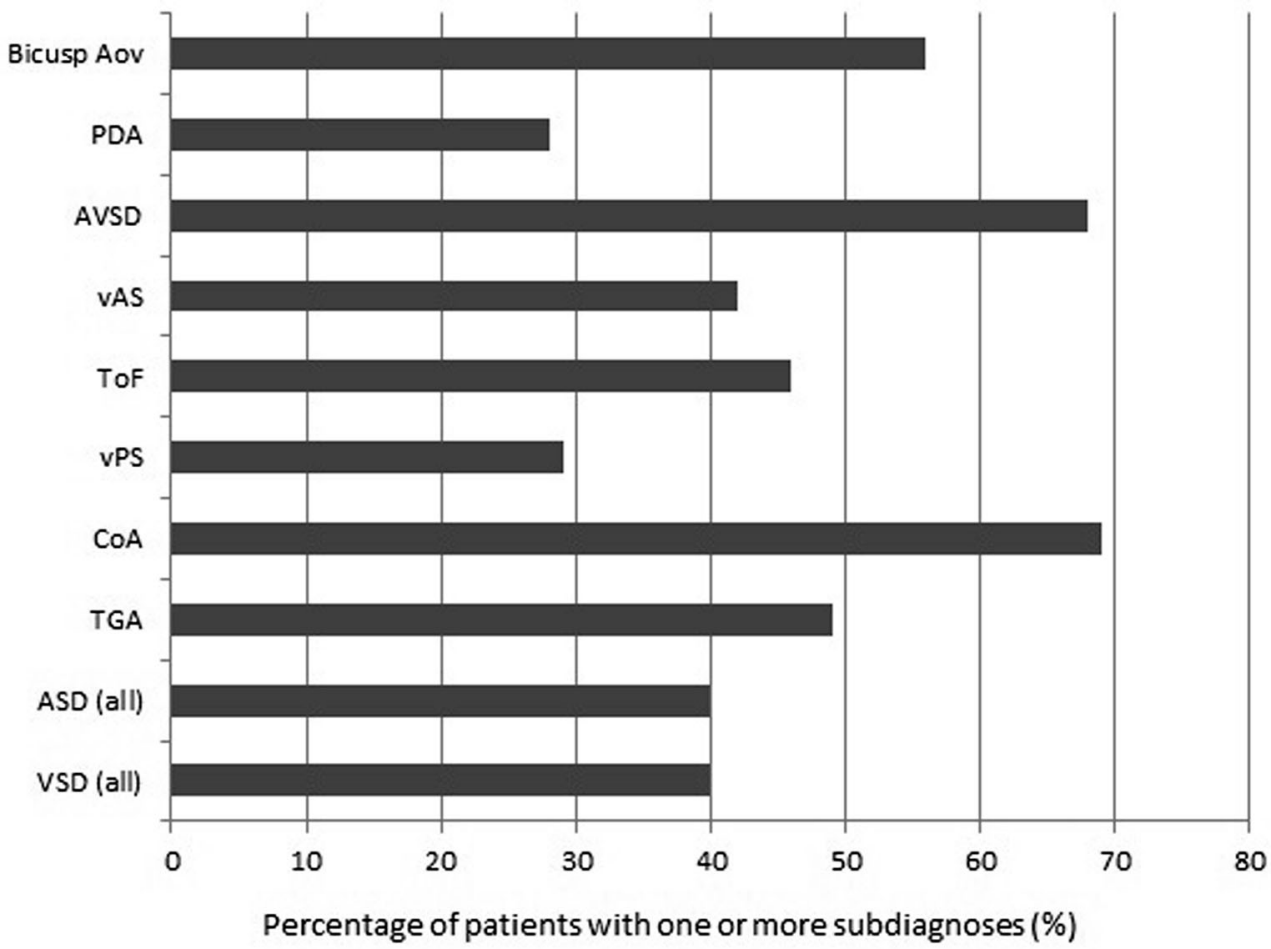
Percentage of patients with one or more subdiagnoses, by main diagnosis. *Biscusp Ao* bicuspid aortic valve, *PDA* persistent ductus arteriosus, *AVSD* atrioventricular septal defect, *vAS* valvular aortic stenosis, *ToF* Tetralogy of Fallot, *vPS* valvular pulmonic stenosis, *Coa* coarction of the aorta, *TGA* transposition of great arteries, *ASD* atrial septal defect, *VSD* ventricular septal defect

One or more subdiagnoses were registered for 3543 patients (42 %). Fig. 3 shows the percentage of patients with one or more subdiagnoses, stratified by a selection of main diagnoses. This percentage was highest in patients with atrioventricular septal defects and coarctation of the aorta in particular (68 and 69 % respectively).

**Fig. 4:**
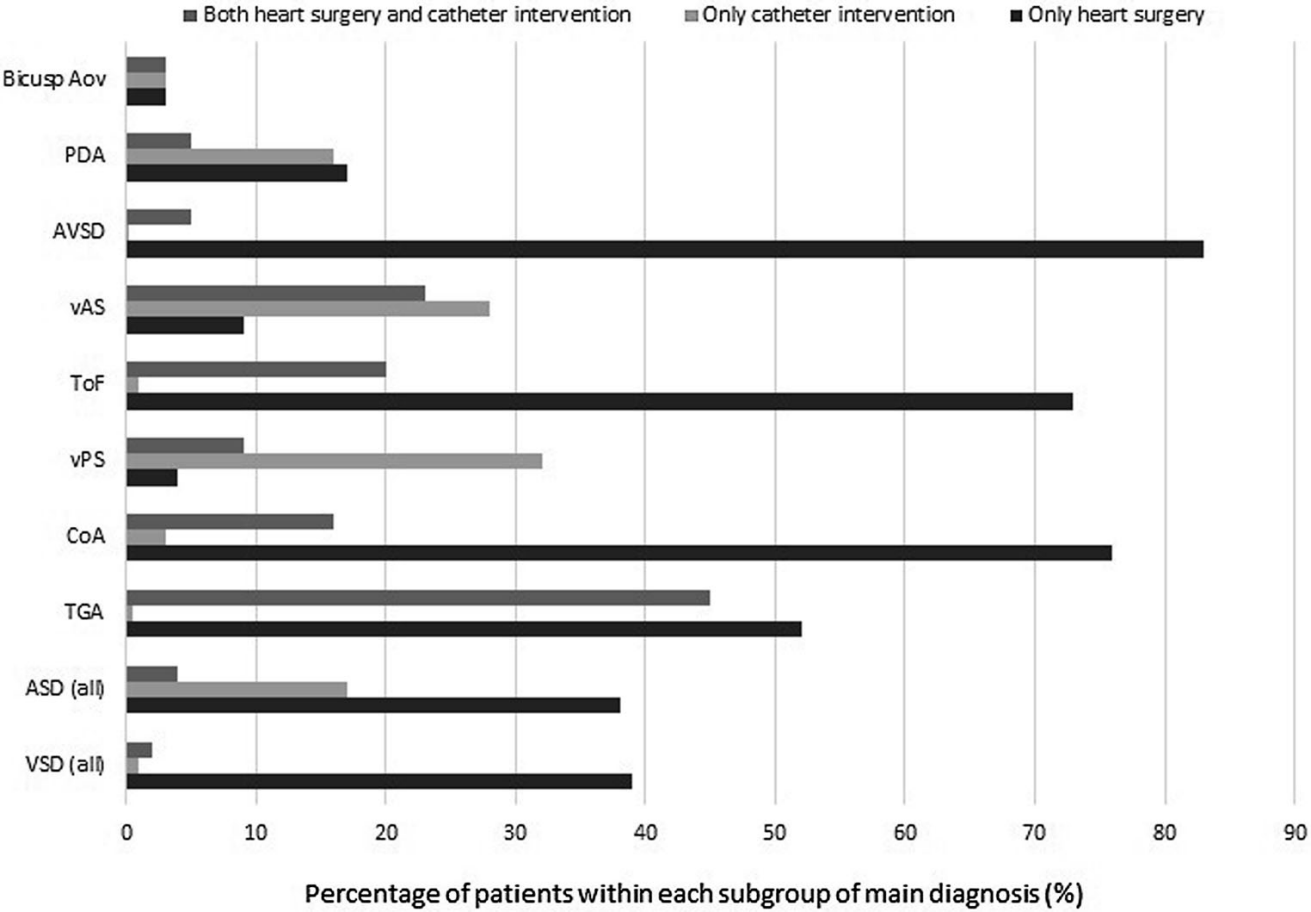
Prevalence of interventions, by main diagnosis. *Biscusp Ao* bicuspid aortic valve, *PDA* persistent ductus arteriosus, *AVSD* atrioventricular septal defect, *vAS* valvular aortic stenosis, *ToF* Tetralogy of Fallot, *vPS* valvular pulmonic stenosis, *Coa* coarction of the aorta, *TGA* transposition of great arteries, *ASD* atrial septal defect, *VSD* ventricular septal defect

Sixty percent of patients had undergone at least one intervention, either catheter intervention or heart surgery. The distribution of interventions, by main diagnosis, is shown in Fig. 4. Patients with transposition of the great arteries, Tetralogy of Fallot, coarctation of the aorta and atrioventricular septal defects had the highest percentage of surgical interventions. Patients with valvular aortic stenosis, transposition of the great arteries and valvular pulmonary stenosis had the highest percentage of catheter interventions.

## Discussion

The KinCor registry has successfully included over 8000 patients so far, and inclusion continues.

Congenital heart disease databases may be important tools for research. However, only few have been available so far. In Denmark [11] and Germany [12] large national registries for CHD are operating that have provided data on, for instance, the risk for psychiatric disorders in CHD patients [13] and on the epidemiology of CHD in Germany [12, 14, 15]. Currently, Eurocat is the largest European Network of population-based registries for congenital anomalies [16–18]. The network consists of 43 registries from 23 European countries. The Netherlands is a member of this European network, but inclusion is limited to the three Northern provinces, representing 10 % of all births in the Netherlands.

Cardiac surgery databases, such as the Congenital Heart Surgery Database of the Society of Thoracic Surgeons [19, 20] and the European Association for Cardio-Thoracic Surgery database [21], include surgery-related data primarily, excluding parts of the paediatric congenital heart population. In contrast, KinCor provides insight into the prevalence, epidemiology and outcome of the full spectrum of paediatric heart disease, with nationwide coverage.

Ventricular septal defect (VSD) was by far the most common morphological congenital heart defect in KinCor (18 %). In contrast, in the Danish Register of Congenital Heart Disease [11] VSD accounted for only 8 %. Compared with the KinCor database, patent arterial duct was more common in the Danish database, accounting for 10.7 % of all CHD. This difference can be explained by the fact that the Danish database included premature infants with patent arterial duct as the only cardiac diagnosis [22]. In the German PAN study the proportion of VSD among all CHD was almost 50 % [12]. This may be due to the fact that approximately 70 % of patients with mild cardiac defects were enrolled by non-academic cardiology units. The PAN study therefore included relatively more mild congenital heart defects. In contrast, the KinCor project has only included patients followed in academic paediatric cardiology departments. Inclusion will start in non-university hospitals soon.

The CONCOR registry [9], the Dutch national registry for adult patients with CHD, has shown results more similar to ours. This may point towards a bias of inclusion of relatively older children in the initial phase of data inclusion in KinCor. Among adults with CHD, Tetralogy of Fallot was the most prevalent main diagnosis (14.1 %) followed by aortic coarctation (10.4 %). Another difference compared with the KinCor registry is that the CONCOR registry includes patients from non-academic hospitals, which may have influenced inclusion rates of patients.

In the Netherlands, CONCOR has set the stage for the successful use of registry data to improve knowledge on CHD in the adult population. The KinCor database was designed to allow close collaboration between these systems. Since the KinCor database uses internationally acknowledged coding systems for diagnosis and interventions, coupling to other international databases, including for example CONCOR, the PAN study or the more recently launched DANARA project [23] should be possible, within the limits set by legislation on privacy and databases.

Databases such as KinCor may provide a platform to identify patients for translational research, e. g. on predictive value of morphological characteristics on outcome (such as other researchers have shown for bicuspid aortic valves [24]), on mechanisms of inheritance or biomarkers, or treatment targets for impending heart failure. In its current stage KinCor is not related to a biobank, mainly for practical reasons. Coupling of KinCor data to existing or new biobanks is an opportunity for the future.

There are some limitations to consider when evaluating the KinCor registry. Firstly, the response rate is currently estimated to be 76 % of eligible. Non-response is a common and an inevitable part of population registries. At present, however, there is no reason to assume underrepresentation of certain diagnoses or age categories because of non-response.

Secondly, patients with complex congenital heart defects are currently labelled according to the main diagnosis. The consequence is that there may be some heterogeneity within each subgroup of patients with heart disease with the same main diagnosis.

In the KinCor database, more boys (55.3 %) than girls (44.7 %) have been registered. A similar sex distribution has been found in the CONCOR database (52 % versus 48 %) [9]. One possible explanation of the male dominance in our patient population may be a higher prevalence of complex congenital heart defects in boys, as previously suggested by data from the United Kingdom [25]. This requires further research.

A small percentage of the patients in the database at present is older than 18 years of age. These are patients that had not yet turned 18 at the start of inclusion in 2012. Retrieval of details of their history and follow-up may not be facilitated by KinCor, since there may no longer be a direct relationship between participating physicians and these adult patients. This may be solved by coupling of KinCor and CONCOR data in the future. At present KinCor, like many other databases in the field, depends on external and temporary funding.

## Conclusion

Currently, the KinCor database contains data of a considerable number of children with heart disease and data of other patients continue to be collected. The KinCor database will provide a unique opportunity for epidemiological research on congenital and other types of heart defects in children in the Netherlands.
